# CrAssphage and its bacterial host in cat feces

**DOI:** 10.1038/s41598-020-80076-9

**Published:** 2021-01-12

**Authors:** Yanpeng Li, Emilia Gordon, Ryan C. Shean, Amanda Idle, Xutao Deng, Alexander L. Greninger, Eric Delwart

**Affiliations:** 1 Vitalant Research Institute, 270 Masonic Avenue, San Francisco, CA 94118 USA; 2 Department of Laboratory Medicine, University of California, San Francisco, CA 94118 USA; 3 The British Columbia Society for the Prevention of Cruelty to Animals, Vancouver, BC V5T 1R1 Canada; 4 Department of Laboratory Medicine and Pathology, University of Washington Medical Center, Seattle, WA 98195 USA

**Keywords:** Metagenomics, Genetic variation, Bacteriophages

## Abstract

CrAssphages are a diverse group of related phages detected in human feces where they are the most prevalent and abundant prokaryotic virus. CrAssphages’ cellular host has been identified as the anaerobic *Bacteroides intestinalis*. CrAssphage has also been reported in non-human primates and environmental samples and has been proposed as a marker of human fecal contamination. Here we describe crAssphage DNA in a feline fecal sample. 95% of the ~ 100 Kb genome could be assembled and classified in genus 1 of the recently proposed Alphacrassvirinae subfamily. The cat origin of the fecal sample was confirmed by partial mitochondrial DNA sequencing. High levels of *Bacteroides intestinalis* DNA could also be detected in this cat’s feces. Fecal samples longitudinally collected over a 4-week period showed the continuous shedding of crAssphage DNA. We therefore report the first genome sequence-confirmed detection of crAssphage in fecal samples of a non-primate mammal.

## Introduction

The circular crAssphage DNA genome of ~ 100 Kb was initially assembled in 2014 from human feces metagenomics data^1^ and is considered the most abundant human gut phage^2^. CrAssphage detection has not been associated with any clinical conditions^3^. The phage’s presence has been tightly associated with human feces and sewage world-wide and has also been detected in feces from non-human primates^3^. CrAssphages are highly diverse and have been classified into 10 genera sharing > 40% of their ORFs^2^. CrAssphage’s bacterial host was initially predicted based on co-occurrence to be a *Bacteroides* sp*.* and then shown to be the anaerobic *Bacteroides intestinalis* in which it replicates without lysis or lysogeny^1,2,4,5^. CrAssphages’ utility as a potential marker for human fecal contamination in sewage and other environmental samples has been proposed and extensively tested^6–11^ and compares favorably to other viral markers such as pepper mild mottle and tobacco mosaic virus^12,13^.

In the process of analyzing a pool of feline feces from an animal shelter using viral metagenomics we identified large crAssphage contigs. In order to follow up on this unexpected find in a non-human sample we performed further deep sequencing and assembled and annotated 95% of a crAssphage genome, confirmed the feline origin of the fecal sample, and identified DNA of its putative bacterial host. We therefore demonstrate the first genome-confirmed detection of crAssphage in the gut of a non-primate animal, here a cat.

## Materials and methods

### Generation of libraries and sequencing

To enrich for viral-like particles (VLP), samples processing and library generation were done according to our previous protocol^14,15^. Briefly, 1 g of feces from each cat was vortexed in 2 ml phosphate buffer saline (PBS) with zirconia beads, and 3–4 samples were further pooled into one tube. Viral particles were then enriched by collecting the supernatant resulting from a 10 min spin at 8000 rpm at room temperature in a table top microfuge which was then filtered through a 0.45-µm filter and digested with a mixture of nuclease enzymes prior to nucleic acid extraction. Nucleic acids were extracted with MagMAX viral RNA isolation kit (Thermo Fisher, USA) and amplified using random RT-PCR^15^. Illumina library was generated using the transposon based Nextera XT Sample Preparation Kit (Illumina, CA, USA) and sequenced on both the MiSeq (2 × 250 bases, dual barcoding) and HiSeq 4000 platform (2 × 150 bases, dual barcoding). To improve the phage genome coverage, the above VLP nucleic acids were also amplified using rolling cycle amplification (RCA) (TempliPhi 500 amplification kit, Sigma-Aldrich, USA) prior to Nextera XT based library generation. Sequencing data generated using both random RT-PCR and RCA methods were used to assemble the genome. To enrich for bacterial DNA, total nucleic acids were extracted using the Qiagen DNA mini kit (Qiagen, Hilden, Germany) from the fecal supernatant without filtration and nuclease treatment steps and Nextera XT used.

### Bioinformatics

An in-house analysis pipeline was used for viral metagenomic analysis^15^. Briefly, duplicate and low-quality reads, adaptors and primer sequences were removed using VecScreen, human and bacterial sequences were then removed using Bowtie^16^. The Ensemble program^17^ was used to assemble contigs and both contigs and singlet reads were then queried against all annotated viral sequences available in GenBank using BLASTx (v.2.2.7). Geneious R11 program was used to align crAssphage reads and contigs to reference viral genome (GenBank NC_024711) and generate the near full-length genome. In order to evaluate the sequence abundance of crAssphage and its host *Bacteroides* in each library, all the sequences from each library were mapped against our near full-length crAssphage genome and *Bacteroides intestinalis* (GenBank NZ_ABJL02000008) using Bowtie with a minimum identity of 0.95, and the relative abundance was normalized as RPM (reads per million). All the short reads sequencing data from different libraries are available at NCBI Sequence Read Archive (SRA) under the BioProject number PRJNA565775 (BioSample accession SAMN16266519-16266527).

### CrAssphage detection, polymerase amplification and mitochondria sequencing

Total nucleic acid was extracted from individual cat fecal samples collected from multiple timepoints using the QIAamp MinElute Virus Spin Kit (Qiagen, Hilden, Germany). The primers used for the detection of crAssphage were: forward 5′-AGACGCGATGAAGAACTGCT-3′ and reverse 5′-CCATCGGGAGCAGTAAGACC-3′; PCR conditions are as follows: 95 °C 3 min, 40 cycles of 95 °C 30 s, 56 °C 30 s and 72 °C 40 s, then a final extension at 72 °C for 7 min. A ~ 590 bp product was visualized on agarose gel. Polymerase B gene was amplified according to a previous method^8^, using forward 5′-CGGCGGGTTAATCAAAATAGAA-3′ and reverse 5′-GCGGAGAACCCCATTTATTAATAAG-3′. In order to sequence mitochondrial sequences in the cat fecal sample, previously described universal primers (without 5′-tagged sequence) that targeted a conserved region of 16 s rRNA gene were used^18^, U16S-F 5′-ACCGTGCAAAGGTAGCATAAT-3′ and U16S-R 5′-TCCGGTCTGAACTCAGATCAC-3′; PCR conditions were as follows: 95 °C 3 min, 40 cycles of 95 °C 30 s, 55 °C 30 s and 72 °C 35 s, with a final extension at 72 °C for 7 min. A ~ 500 bp product was visualized on agarose gel, purified and directly Sanger sequenced with a commercial vendor using an Applied Biosystems capillary sequencer. The ABI electrophoregram file was checked using Geneious R11.

### Phylogenetics for genotyping crAssphage

The full-length polymerase B protein sequences including ten representative sequences were aligned using the Muscle program in MEGA 7.0; phylogenetic trees were inferred using Maximum-Likelihood method. Model test module of MEGA 7.0 was used to determine the best substitution model. A phylogenetic tree of polymerase protein sequences was generated using the bootstrap method (1000 times) under Le_Gascule_2008 model (LG) with frequencies and gamma distributed, invariant sites (G + I) model.

## Results

### Metagenomics detection

**Figure 1:**
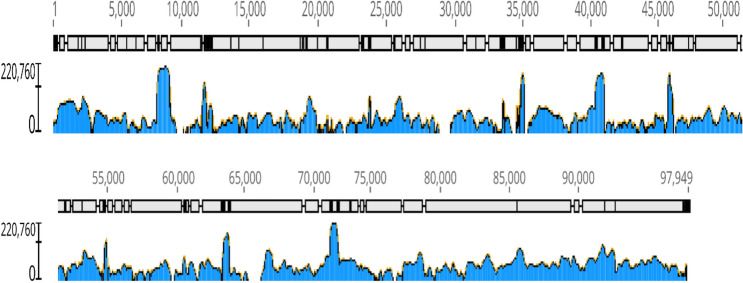
Genome coverage of the feline feces crAssphage genome (MW063138).

**Figure 2:**
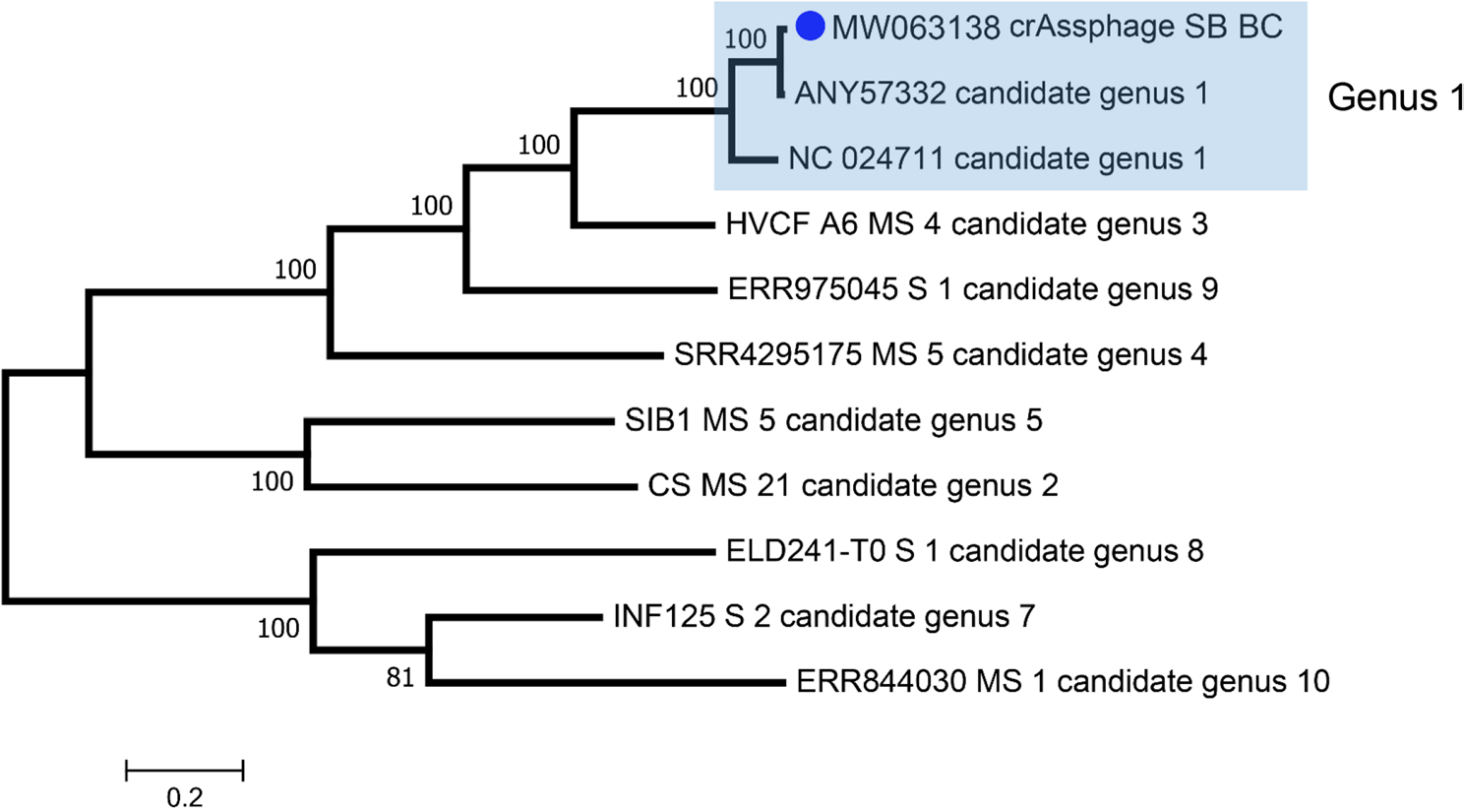
Phylogenetic analysis using maximum likelihood method based on the complete amino acid sequence of the polymerase protein. Representative crAssphage genomes from different genera found in human feces were included.

During an investigation of an unexplained feline vomiting outbreak in an animal shelter we used viral metagenomics to identify potentially pathogenic viruses. Four mini-pools of 3 feline fecal samples each (total of 12 cats) were initially analyzed using viral metagenomics. Following VLP enrichment RNA and DNA were purified, reverse transcribed, randomly amplified, converted to Illumina compatible DNA, and deep sequenced on the Illumina MiSeq platform (SAMN16266519-16266522). Reads were de novo assembled and then analyzed using BLASTx for their ability to encode viral proteins (see “Materials and methods”). One pool (#4) consisting of 3 fecal samples from sick cats yielded multiple long contigs with high levels of nucleotide identity to crAssphage genome. In order to improve coverage of the crAssphage genome each of the 12 individual cat fecal samples were individually extracted and tested for crAssphage DNA using PCR designed from an initial crAssphage-like contig. A single fecal sample in pool #4 from cat SB2894 collected December 2, 2019 was positive. SB2894, a 5-year old male neutered domestic medium hair cat, was in the animal shelter from December 2nd 2019 to January 4th 2020 except for a 6-day period from December 11–17th when he was in a home. That individual fecal sample was further analyzed using the same protocol of random RT-PCR of VLP-associated nucleic acids and deep sequenced. Pool 4 was also re-sequenced on the MiSeq and in order to generate even more viral reads the same cat SB2894 random RT-PCR VLP library was also sequenced on a HiSeq4000 (SAMN16266525). Lastly in order to focus the sequencing on DNA alone enriched VLP associated nucleic acids from SB2894 and pool 4 were amplified using PhiX174 DNA polymerase (rather than random RT-PCR) using random primers in a multiple displacement amplification (MDA) followed by use of the Illumina Nextera XT prior to deep sequencing using the HiSeq4000 platform. All crAssphage reads from these 6 libraries containing crAssphage reads (from pool 4 and SB2894) were then used to map against a crAssphage genome (Genbank: NC_024711) yielding a ~ 95% complete genome (GenBank: MW063138). Because of gaps in the polymerase B gene typically used for crAssphage phylogenetic analysis this region was PCR amplified, Sanger sequenced, incorporated in the final genome, and used for phylogenetic analysis. The crAssphage genome coverage is shown in Fig. 1. Phylogenetic analysis with representative sequences of the different genera of crAssphage recently described^4,8^ was then performed showing that the cat feces crAssphage belonged to genus 1 (Fig. 2). The overall nucleotide identity between feline crAssphage strain SB2894 and its closest relative, an uncultured crAssphage from human feces collected in the USA on 15 July 2010 (GenBank: NC_024711) was 88% or 93.6% if only measuring aligned regions (i.e. discounting gaps due to missing sequence).

**Figure 3:**
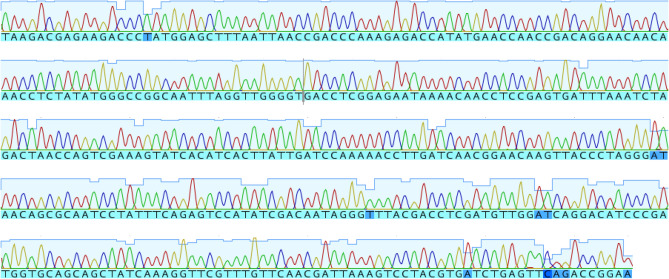
Sanger sequencing of segment of the *Felis catus* (Cat) 16S ribosomal RNA gene. Single (not mixed) electrophoregram peaks indicate absence of major contamination from other 16S rRNA sequences.

In order to confirm that the deep sequenced crAssphage positive fecal sample was indeed from a cat, a phylogenetically informative ~ 500 bp region of the mitochondrial 16S rRNA was PCR amplified and sequenced. The amplicons yielded a 100% match to cat mitochondria with no evidence of a mixture of mitochondria in the Sanger electropherogram (Fig. 3).

**Figure 4:**
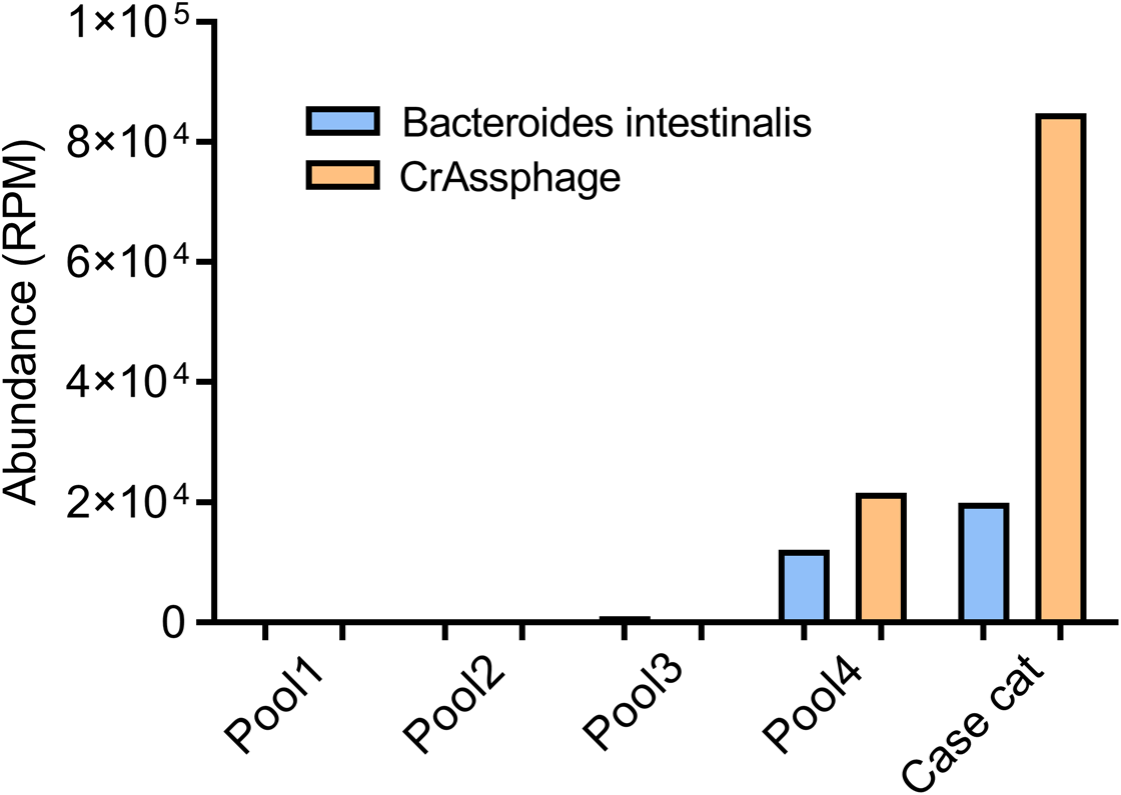
Abundance (calculated as RPM) of crAssphage and *Bacteroides intestinalis*. Libraries of cat feces pools were generated by random RT-PCR amplification of VLP nucleic acids. For the case cat, total fecal supernatant nucleic acids were extracted without filtration and nuclease treatment steps and processed using RCA. Illumina libraries were then generated using Nextera XT and sequenced on a MiSeq. Bowtie was used to then quantify reads of crAssphage and *Bacteroides intestinalis*.

We next tested whether the expected bacterial host of crAssphage could be detected in feces from SB2894. The *Bacteroides intestinalis* genome (GenBank NZ_ABJL02000008) was used to identify matching reads (using the program Bowtie requiring a match of ≥ 95% identity). Of the four initial fecal pools only pool 4 showed a high level of *Bacteroides intestinalis* related sequences with ~ 2 × 10^4^ reads per million total reads or 2%. Because these initial libraries were generated to enrich viral nucleic acids, another library was made using total DNA content from the implicated fecal sample (“Materials and methods”). When the reads from that library were again analyzed for *Bacteroides intestinalis* DNA sequences an even higher RPM of matching reads could be mapped to that bacterial genome (Fig. 4). We therefore conclude that the feces of cat SB2894 contained a significant level of *Bacteroides intestinalis*.

We next tested whether crAssphage DNA could be detected in multiple fecal samples longitudinally collected over a period of one month from case cat SB2894. CrAssphage DNA could be amplified by PCR in 9/10 of these fecal samples (Table 1). This result indicated that crAssphage was chronically present in the gut of this cat during the one month sampling period.

**Table 1:** Serial crAssphage detection from cat SB2894.

ID	Dec2	Dec19	Dec20	Dec21	Dec23	Dec24	Dec25	Dec28	Jan1	Jan2
SB2894	+	+	+	−	+	+	+	+	+	+

### Discussion

The crAssphage genome reported here belonged to genus 1, the most common clade reported in human feces^2^. Whether the *Bacteroides intestinalis* and crAssphage strains detected in SB2894 were transmitted from human contacts is unknown. Longitudinal sampling of human feces have shown that crAssphage shedding can be either prolonged, lasting up to several months, or more intermittent^3^.

CrAssphage DNA detection and its quantitation has been extensively examined as a proxy for human fecal contamination^6–11^. Stachler et al. used 384 different primer pairs to identify the most human specific and sensitive crAssphage PCR primer pairs when compared to animal samples^6^. Of 222 animal feces (Canadian geese, dogs, cows, gulls, horses, elks, chickens, pigs, beavers, and deer) tested with the two most human-specific primer pairs, amplification was detected in only 2/25 (8%) gulls and 1/41 (2.4%) dog feces yielding a human-specificity of 98.6%^6^. Another PCR study using the same primers on 177 animal samples (bird, cat, cattle, deer, dog, emu, goat, horse, kangaroo, koala, pig, sheep, waterfowls) detected positive PCR signal(s) in 2/12 cattle abattoir wastewater samples and 10/14 cats at viral loads generally 4 to 5 log10 GC/L (genome concentration per liter) lower than in human feces^10^. When one of the PCR primer sets was used on 360 non-human fecal samples from 14 species from Australia (bird, cat, cow, chicken, deer, dog, emu goat, horse, kangaroo, pig, sheep, waterfowl) only 5/30 cats yielded a positive PCR^19^. Another test of 73 animal feces (alligator, bird, cat, cattle, deer, dog, duck, horse, poultry) with a different set of crAssphage PCR primers was performed and only the poultry litters (6/10) were positive for crAssphage DNA^11^. A further 78 animals tested (cow, sheep, horse, pig, and rhesus macaque, rat, and cat from laboratory sources) were all crAssphage DNA PCR negative^8^. Raw human sewage, 15 cattle feces and 3 pig wastewaters from Japan were tested and only raw human-impacted sewages and pig waste waters were crAssphage DNA PCR positive^12^. These studies therefore showed that crAssphage is common in human feces and human-impacted sewage. Nonetheless occasional crAssphage DNA PCR detection in feces from cats, dogs, gulls, cattle and pig waste water and poultry indicated that crAssphage may not be limited to human guts. The PCR detection of crAssphage in the animal testing studies mentioned above were not confirmed by PCR amplicon sequencing nor metagenomic genome sequencing.

We report the first near complete crAssphage genome derived from a non-human (feline) fecal source. Feces from that cat also contained *Bacteroides intestinalis*, the cellular host of crAssphage^1,2,4,5^. Phage DNA could be amplified from feces collected over several weeks after the cat entered an animal shelter indicating that coprophagy of human feces (a rare occurrence for cats) just prior to entry into the animal shelter could not explain its detection. Whether cats commonly contain *Bacteroides intestinalis* and crAssphage in their gut is currently unknown but this and prior PCR based studies indicate that infections of cats may be common. The possible origin of crAssphage in cats from their human companions will require further studies.

The detection of crAssphage DNA in feces from a cat also indicates that the use of crAssphage DNA as a strictly human marker of human fecal contamination may need to be expanded to include other mammalian species occasionally hosting *Bacteroides intestinalis*.

## Data Availability

All sequencing data are available at NCBI Sequence Read Archive (SRA) as mentioned in the “Materials and methods”.
